# The Prevalence of Metabolic Syndrome in Ethiopian Population: A Systematic Review and Meta-analysis

**DOI:** 10.1155/2020/2701309

**Published:** 2020-12-16

**Authors:** Sintayehu Ambachew, Aklilu Endalamaw, Abebaw Worede, Yalewayker Tegegne, Mulugeta Melku, Belete Biadgo

**Affiliations:** ^1^Department of Clinical Chemistry, School of Biomedical and Laboratory Sciences, College of Medicine and Health Sciences, University of Gondar, Gondar, Ethiopia; ^2^Departement of Pediatrics and Child Health Nursing, School of Nursing, College of Medicine and Health Sciences, Bahir Dar University, Bahir Dar, Ethiopia; ^3^Department of Parasitology, School of Biomedical and Laboratory Sciences, College of Medicine and Health Sciences, University of Gondar, Gondar, Ethiopia; ^4^Department of Hematology and Immunohematology, School of Biomedical and Laboratory Sciences, College of Medicine and Health Sciences, University of Gondar, Gondar, Ethiopia

## Abstract

**Background:**

The metabolic syndrome is a clustering of hyperglycemia/insulin resistance, hypertension, dyslipidemia, and obesity which are risk factors for cardiovascular disease, type 2 diabetes and stroke, and all-cause mortality. The burden of metabolic syndrome is emerging alarmingly in low- and middle-income countries such as Ethiopia; however, there is lack of comprehensive estimation. This study aimed to determine the pooled prevalence of metabolic syndrome in Ethiopia.

**Methods:**

This systematic review and meta-analysis included original articles of observational studies published in the English language. Searches were carried out in PubMed, Google Scholar, and Africa Journals from conception to August 2020. A random-effects model was used to estimate the pooled prevalence of metabolic syndrome in Ethiopia. Heterogeneity was assessed using the *I*^2^ statistic. Subgroup analysis was also conducted based on sex/gender and study subjects. Egger's test was used to assess publication bias.

**Results:**

Electronic and gray literature search retrieved 942 potentially relevant papers. After removing duplicates and screening with eligibility criteria, twenty-eight cross-sectional studies were included in this meta-analysis. The pooled prevalence of metabolic syndrome in Ethiopia was found to be 34.89% (95% CI: 26.77, 43.01) and 27.92% (95% CI: 21.32, 34.51) by using NCEP/ATP III and IDF criteria, respectively. The weighted pooled prevalence of metabolic syndrome was higher in females 36.74% (95% CI: 20.72, 52.75) and 34.09% (95% CI: 26.68, 41.50) compared to males 22.22% (95% CI: 14.89, 29.56) and 24.82% (95% CI: 18.34, 31.31) by using IDF and NCEP/ATP III criteria, respectively. Subgroup analysis based on the study subjects using NCEP/ATP III showed that the weighted pooled prevalence was 63.78%(95% CI: 56.17, 71.40), 44.55% (95% CI: 30.71, 52.38), 23.09% (95% CI: 19.74, 26.45), 20.83% (95% CI: 18.64, 23.01), and 18.45% (95% CI: 13.89, 23.01) among type 2 diabetes patients, hypertensive patients, psychiatric patients, HIV patients on HAART, and working adults, respectively. The most frequent metabolic syndrome components were low HDL-C 51.0% (95% CI: 42.4, 59.7) and hypertriglyceridemia 39.7% (95% CI: 32.8, 46.6).

**Conclusions:**

The findings revealed an emerging high prevalence of metabolic syndrome in Ethiopia. Therefore, early intervention is required for the primary prevention of the occurrence of metabolic syndrome and the further reduction of the morbidity and mortality related to it.

## 1. Background

Metabolic syndrome (MetS) is a cluster of interrelated risk factors that have been associated with cardiovascular disease (CVD), stroke, diabetes mellitus, and other comorbidities [1, 2]. Insulin resistance, obesity, dyslipidemia, and hypertension are considered to be the primary components of MetS [3, 4]. The worldwide prevalence of MetS and noncommunicable chronic diseases in the adult population is on the rise [5]. It has been estimated that the prevalence of MetS ranges 20–25% of the adult population globally [6]. The epidemiologic nature of MetS is also emerging alarmingly and being common in Africa in contrast to the earlier trend of being considered rare. The increase in the prevalence of MetS in Africa is thought to be due to divergence from traditional African to Western lifestyles [7]. High prevalence of MetS has been reported in some sub-Saharan Africa countries such as in Morocco (16.3%) and South Africa (33.5%) [8].

The individual with MetS has 2-3 times higher chance of developing stroke and CVD than without MetS [9, 10]. It has also a six-fold greater risk of developing type 2 diabetes (T2DM) [11]. T2DM has become one of the major causes of premature illness and death, mainly through the increased risk of CVD disease [12]. MetS is also associated with other comorbidities such as cancer, nonalcoholic fatty liver disease, and other reproductive disorder [13–15]. It is also suggested that mortality due to MetS is more than twice that without the syndrome [16]. The prevalence of MetS varies across different populations. MetS appear to be more common in the presence of comorbidities such as diabetes mellitus, hypertension, and HIV infection than their counterparts. Around 85% of those with diabetes have MetS in the United States in contrast to 25% of working adults in Europe and Latin America [17, 18]. Likewise, the high prevalence of HIV infection in developing countries and concomitant antiretroviral therapy is also associated with the rise of MetS [19]. Todowede O. O. et al. have reported in a meta-analysis that MetS prevalence among people living with HIV was 21.5% in contrast to uninfected 12.0% [20]. MetS rates are rising in developing countries in an epidemic situation [7]. For instance, in Ethiopia, the prevalence of MetS among T2DM, hypertensive, and HIV patients is estimated to be high [21–23]. The pathogenesis of the MetS is multidirectional, but obesity-induced insulin resistance is the major pathway for the occurrence of MetS [24]. Similarly, increased risk of MetS and its components is attributed to antiretroviral therapy and other related factors [25]. Sociodemographic factors, behavioral factors, stress, family history of disease, and some anthropometric measurements have been reported as associated factors of MetS in different literatures [26–28]. For instance, a study conducted among Malaysian population has showed that increasing age and low physical activity have been associated with increased odds of MetS [29].

Due to the dramatic increase in urbanization, smoking, severe stress-related problems of poverty, nutrition transition, reduced physical activity, and over nutrition increasing on top of the already high prevalence of undernutrition, sub-Saharan Africa is facing a rapid escalation of MetS and noncommunicable chronic diseases and associated mortality [30–32].

In Ethiopia, several studies were conducted to assess the prevalence of MetS among different study subjects having great disparity and inconsistent findings. However, there is lack of comprehensive estimation of MetS in Ethiopia. Hence, this systematic review aimed to determine the pooled prevalence of MetS among the Ethiopian population of various study subjects. This will provide the necessary information for policymakers, clinicians, and concerned stakeholders in the country to provide an appropriate strategy and intervene in the control, prevention, and management of MetS.

## 2. Methods

### 2.1. Protocol and Search Strategy

This systematic review and meta-analysis was reported according to the Preferred Reporting Items for Systematic Reviews and Meta-Analyses statement guideline. The study protocol was registered in the PROSPERO International Prospective Register of Systematic Reviews (CRD42018090944) [33]. An inclusive literature search was conducted to identify studies about the prevalence of MetS reported among the Ethiopian population of various study subjects. Both electronic and gray literature searches were carried out systematically. PubMed, African journal of Medline, and Google Scholar were used to retrieve data. The search terms were used separately and in combination using Boolean operators like “OR” or “AND.” An example of keywords used in PubMed to select relevant studies was as follows: [((Metabolic syndrome [Title/Abstract]) OR (MS [Title/Abstract]) OR (MetS [Title/Abstract]) OR (Insulin resistance syndrome [Title/Abstract]) OR (syndrome X [Title/Abstract]) OR (MtS [Title/Abstract])) AND (Ethiopia [Title/Abstract])]. Moreover, a snowball search was used to search the citation lists of included studies. The search incorporated studies recorded up to the 30^th^ of August 2020. The software EndNote version X7 (Thomson Reuters, New York, NY) was used to manage references and remove duplicated references.

### 2.2. Design

This systematic review and meta-analysis was conducted to estimate the pooled prevalence of MetS based on the International Diabetes Federation [6] and National Cholesterol Education Program–Adult Treatment Panel III (NCEP/ATP III) criteria [34] (Table 1) among different study subjects in Ethiopia.

### 2.3. Exclusion and Inclusion Criteria

Observational studies (all of them were cross-sectional studies) that described the prevalence of MetS among the indigenous Ethiopian population were included. All included studies were original research articles published in English and contained the minimum information (study participants and number of MetS cases). Moreover, studies in which MetS has been reported using (i) IDF criteria and/or (ii) NCEP/ATPIII were included. The full text of studies meeting these criteria was retrieved and screened to determine its eligibility. Whereas studies in which MetS was described on other than the Ethiopian population, nonoriginal research (such as review, editorial, and a letter or commentary), and unknown/unclear methods of how MetS was diagnosed were not included.

### 2.4. Study Selection and Quality Assessment

Two reviewers (S.A. and M.M.) independently screened the titles and abstracts in the abovementioned databases to consider the articles in the full-text review. The quality of the studies was appraised using Joanna Brigg's Institute quality appraisal criteria (JBI) [35]. All selected articles were evaluated by using the JBI appraisal checklist. Studies that got 50% and above on the quality scale were considered to have good quality (Table 2).

### 2.5. Data Extraction

To collect relevant data, a data extraction tool was developed, and one reviewer (SA) was responsible for the extraction of data from the included studies. Information regarding authors, year of publication, the study population, type of study/study design, number of participants, gender, diagnostic criteria for defining Mets, region, study area, sampling techniques, and the prevalence of MetS in each study were collected. The extracted data were checked by the second reviewer (AE) for its accuracy and consistency. A third reviewer (YT) was also engaged where necessary.

### 2.6. Data Analysis

The extracted data were entered into Microsoft Excel and analyzed using STATA version 14 (StataCorp. 2009. Stata Statistical Software. College Station, TX: StataCorp LP). A random-effects model was used to obtain an overall summary estimate of the prevalence across studies. Point estimation with a confidence interval of 95% was used. Sensitivity analysis was conducted to assess the role of each study in the final result by excluding each study one by one. The presence of publication bias was assessed by using Egger's test. Trim and fill method analyses have been conducted to obtain a bias-adjusted effect estimate. Heterogeneity across studies was checked by Cochran's *Q* statistic and *I*^2^ statistics. Moreover, meta-regression has been conducted that represent linear predictions for the MetS prevalence as a function of published year. Subgroup analysis was performed based on sex/gender, and study subjects since there have unexplained significant heterogeneity.

## 3. Result

### 3.1. Characteristics of Included Studies

**Figure 1:**
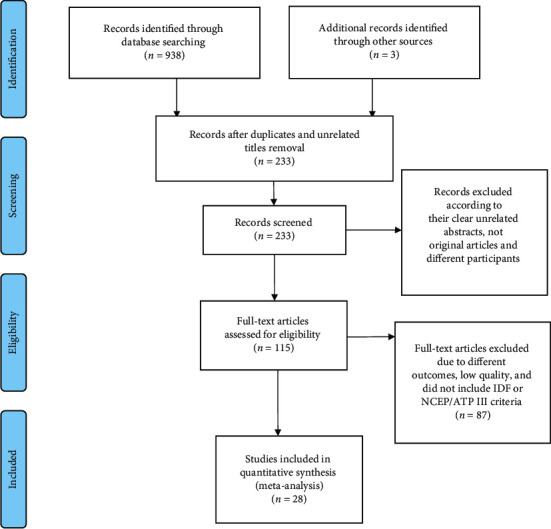
Flow diagram describing the steps of article inclusion to this systematic review.

Out of 942 potential articles, 233 articles were found to be relevant to the topic of interest screened by title and abstract. Then, 115 were found to be eligible for full-text assessment. Of these full-text screened articles, 28 of them comprising 20652 study participants were found to be eligible for meta-analysis (Table 3).  Figure 1 showed the results of the search and reasons for exclusion during the study selection process.

The prevalence of MetS was estimated based on the IDF and NCEP/ATPIII criteria among the Ethiopian population of various study subjects. Fourteen studies reported the prevalence of MetS based on both IDF and NCEP/ATPIII criteria [36–44, 48–50, 54, 56], seven studies based on NCEP/ATPIII criteria only [57–63], and again seven studies by IDF criteria only [45–47, 51–53, 55]. Table 3 presents the characteristics and outcomes of the reviewed studies.

### 3.2. Prevalence of MetS Using IDF and NCEP ATP III Criteria

**Figure 2:**
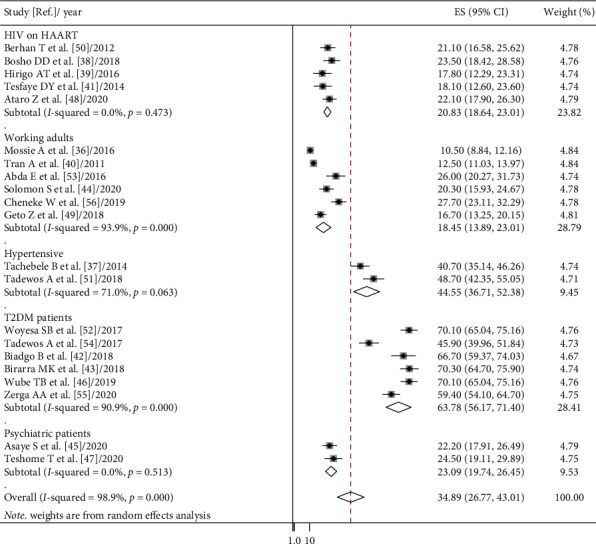
Forest plot of metabolic syndrome prevalence in Ethiopia based on NCEP/ATP III criteria.

**Figure 3:**
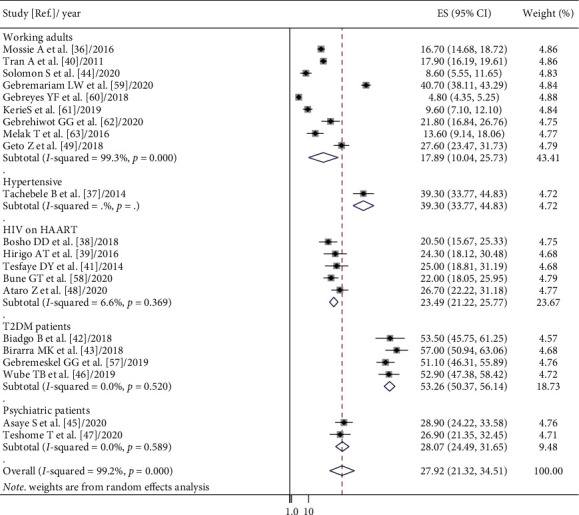
Forest plot of metabolic syndrome prevalence in Ethiopia based on IDF criteria.

The random-effects model was applied since the heterogeneity index of the studies was significant. The pooled prevalence of metabolic syndrome in Ethiopia was found to be 34.89% (95% CI: 26.77, 43.01) and 27.92% (95% CI: 21.32, 34.51) by using NCEP/ATP III and IDF criteria, respectively. Subgroup analysis based on the study subjects using NCEP/ATP III showed that the weighted pooled prevalence was 63.78% (95% CI: 56.17, 71.40), 44.55% (95% CI: 30.71, 52.38), 23.09% (95% CI: 19.74, 26.45), 20.83% (95% CI: 18.64, 23.01), and 18.45% (95% CI: 13.89, 23.01) among type 2 diabetes patients, hypertensive patients, psychiatric patients, HIV patients on highly active antiretroviral therapy (HAART), and working adults, respectively. Using IDF criteria, subgroup analysis based on the study subjects showed that the weighted pooled prevalence was 53.26% (95% CI: 50.37, 56.14), 39.30% (95% CI: 33.77, 44.83), 20.07% (95% CI: 24.49, 31.65), 23.49% (95% CI: 21.22, 25.77), and 17.89% (95% CI: 10.04, 25.73) among type 2 diabetes patients, hypertensive patients, psychiatric patients, HIV patients on HAART, and working adults, respectively (Figures 2 and 3). The weighted pooled prevalence of metabolic syndrome was higher in females 36.74% (95% CI: 20.72, 52.75) and 34.09% (95% CI: 26.68, 41.50) compared to males 22.22% (95% CI: 14.89, 29.56) and 24.82% (95% CI: 18.34, 31.31) by using IDF and NCEP/ATP III criteria, respectively (Table 4).

**Figure 4:**
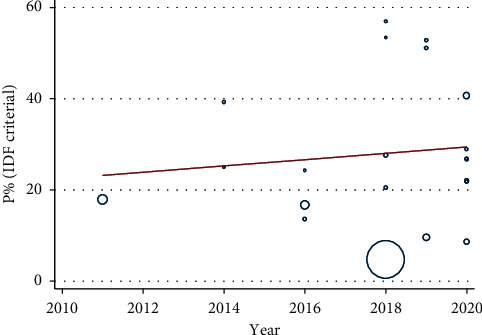
Meta-regression analysis based on published year using IDF criteria.

**Figure 5:**
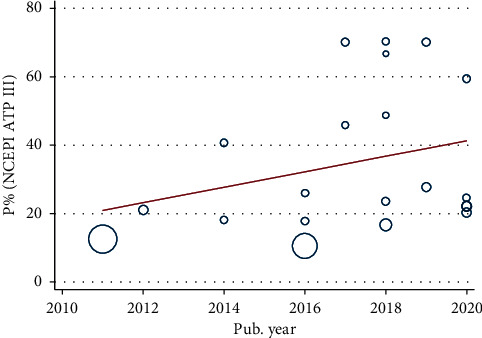
Meta-regression analysis based on published year using NCEP/ATP III criteria.

Moreover, meta-regression has been conducted that characterize the linear predictions for the prevalence of MetS as a function of the increment of published year (Figures 4 and 5). An increasing trend in pooled prevalence of MetS is observed during the 2010–2020 time period in both IDF and NCEP/ATP III criteria.

### 3.3. Prevalence of the MetS Components

The prevalence of the individual components of MetS among the Ethiopian population varied considerably between studies (Table 5). In 22 studies, the overall prevalence of individual components of MetS was reported, whereas in 6 studies, the prevalence of individual components of MetS was not reported. The overall weighted pooled prevalence by the component was as follows: abdominal obesity 35.85% (95% CI: 28.9, 42.8), hyperglycemia 26.4% (95% CI: 20, 32.8), hypertension 27.87% (95% CI: 23.4, 32.2), hypertriglyceridemia 39.7% (95% CI: 32.8, 46.6), and low HDL-C 51.0 (95% CI: 42.4, 59.7) are summarized in Table 5.

### 3.4. Sensitivity Analysis

**Figure 6:**
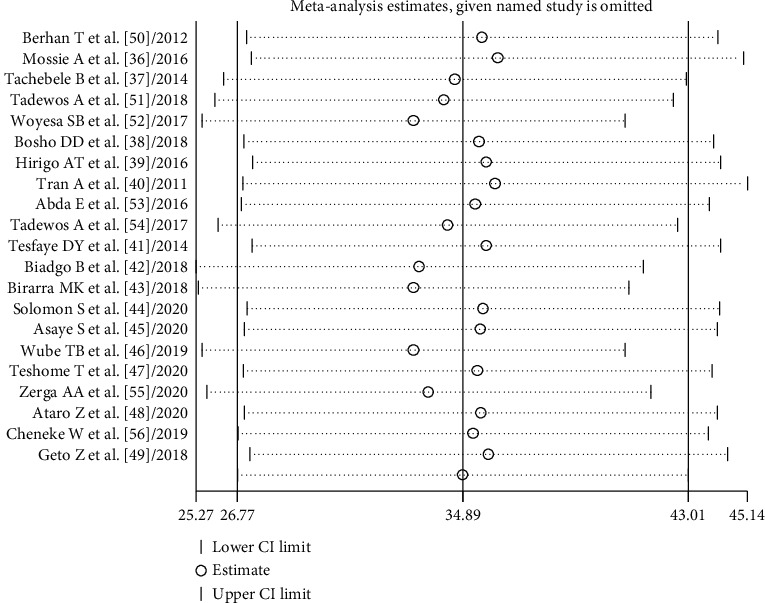
Sensitivity analysis based on NCEP/ATP III criteria.

**Figure 7:**
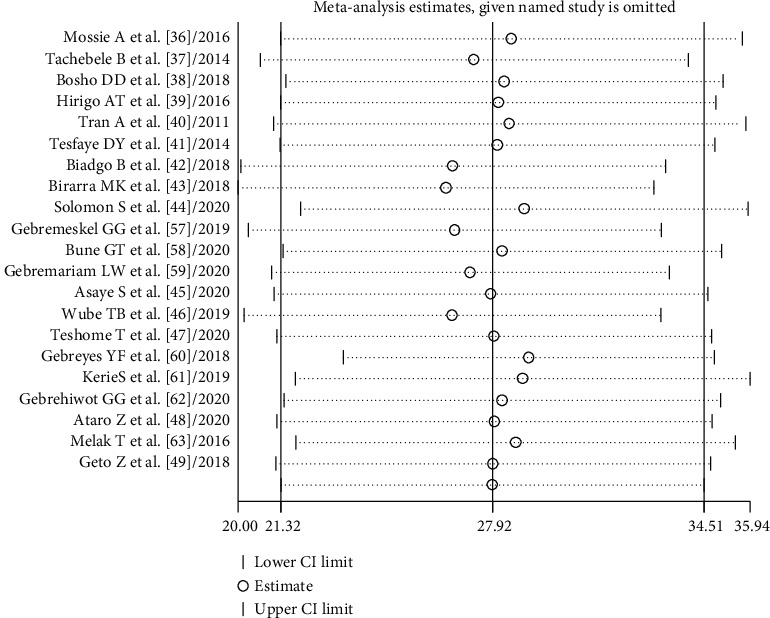
Sensitivity analysis based on IDF criteria.

Sensitivity analysis was performed to assess the effect of each study on the pooled estimated prevalence of MetS by excluding each study step-by-step from the analysis process based on the two given diagnostic criteria (IDF and NCEP/ATP III). The result showed that excluded studies led to no significant changes in the shared estimation of the prevalence of MetS (Figures 6 and 7).

### 3.5. Publication Bias

**Figure 8:**
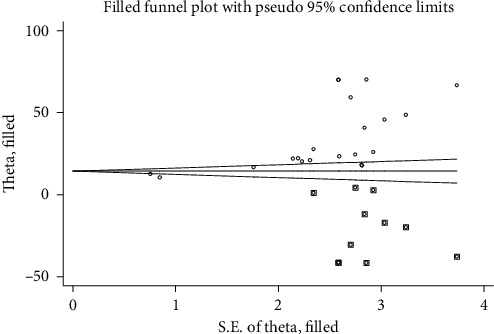
Trim and fill analysis based on NCEP/ATP III criteria.

**Figure 9:**
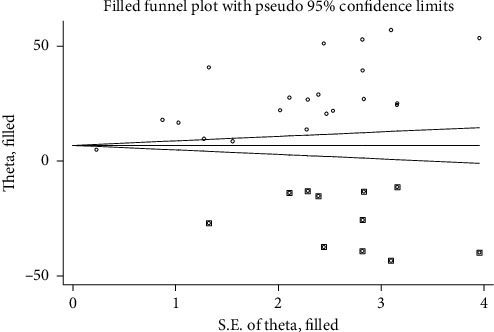
Trim and fill analysis based on IDF criteria.

The included studies were assessed for potential publication bias using Egger's test. Separate analyses using Egger's test based on IDF and NCEP/ATPIII criteria (*p* values were 0.001 and 0.001, respectively) indicated the presence of publication bias. This indicates that the unpublished findings might have shown a larger magnitude of MetS. Adjusting the findings using the trim and fill method would provide a bias-adjusted effect estimate. Therefore, to do so, we have conducted trim and fill method analysis. A bias-adjusted effect estimate of MetS showed 9.67 % (95% CI: 3.16, 16.19) and 15.67% (95% CI: 6.79, 24.55) by using IDF and NCEP/ATPIII criteria, respectively, assuming there are missing studies (Figures 8 and 9). However, there was somehow a difference compared with our previous results indicating minimally impacted by publication bias.

## 4. Discussion

The current systematic review provides evidence of an estimated pooled prevalence of MetS among various study subjects of the Ethiopian population. According to this review, the combined pooled prevalence of MetS was 34.89% (95% CI: 26.77, 43.01) and 27.92% (95% CI: 21.32, 34.51) by using NCEP/ATP III and IDF criteria, respectively. These results were slightly in line with the reports in Brazil (29.6%) [64], Bangladesh (30%) [65], Iran 31% [66], and the United States (33%) [67]. But the current pooled estimate was also higher than the prevalence estimated in Ghana (12.4%) [68], Philippine (11.9%) [69], and the global estimate (20–25%) [6]. The findings were also lower as compared to Palestine (37.0%) [70], the Greek population (43.4%) [71], and the population of Nepal (52.7%) [72]. The variation in reports might be due to the difference in the underling important risk factors such as urbanization, westernization of lifestyle including unhealthy diet and physical inactivity [73], mental stress due to economic, social, and cultural factors [74], genetics, proinflammatory condition [75], intrauterine growth retardation, and over nutrition increasing on top of the already high prevalence of under nutrition [76].

The prevalence of MetS was high (34.89%) when NCEP/ATPIII criteria was used compared to IDF criteria (27.92%) in the current meta-analysis. These might be due to the relative flexibility of the NCEP/ATPIII in which central obesity is not the absolutely required criterion, unlike IDF [77]. However, it was different from a systematic review conducted among the Ghanaian population using these two criteria, NCEP/ATPIII (12.4%) and IDF criteria (21.2%) [68]. The discrepancy between the results of different studies could be attributed to the difference in abdominal obesity and waist circumference in different populations.

We observed high between-study heterogeneity across studies. In the subgroup analysis, the pooled prevalence of MetS using NCEP ATP III criteria was 63.78% among T2DM patients which was higher than the result of the rest of the study subjects, 18.45%, 20.83%, 23.09%, and 44.55% among working adults, HIV patients on HAART, psychiatric patients, and hypertensive patients, respectively. This is also the same using IDF criteria. Though hyperglycemia is one of the components of Mets that increased its prevalence, insulin resistance-linked obesity might be the aggravated reason for the high prevalence of MetS among T2DM patients. In T2DM patients, particularly when glycemic control is poor, lipoprotein lipase activity is reduced [78]. The reduced lipoprotein lipase activity added with poor dieting and lack of regular exercise might be the reason for the higher prevalence of MetS among T2DM patients [79].

In line with other reviews [66, 80], the current meta-analysis demonstrated that the prevalence rate of MetS was higher in females compared to that in males. This has been shown in all the Middle Eastern countries, and the prevalence was much higher among women than men [81]. The higher prevalence of MetS among the women is attributed to abdominal obesity, which is mainly due to low physical activity, higher birth rate, presence of estrogen receptors, and menopause [82]. Besides genetic variation, lifestyle might be the reason.

Low HDL-C was the most frequent individual component of MetS in the current meta-analysis which was similar to reports in Bangladesh (89%) [65], Colombia (62.9%) [18], and Venezuela (58.6%) [83]. However, hypertriglyceridemia was shown the second most prominent MetS component to the contrary high blood pressure report in Bangladesh (30%) [65]. The low concentration of HDL-C favors the accumulation of low-density lipoprotein in the blood vessels greatly due to its poor scavenging capacity of low-density lipoprotein from the body. The coexistence of hypertriglyceridemia and low HDL-C are risk factors for the development of CVD. It is well-known that a low concentration of the HDL-C level is a strong independent risk factor for CVD [84]. As a limitation to this review, a point needs to be noted that there is no uniformity of MetS definitions and waist circumference cutoffs points. Besides, high heterogeneity has been observed. Furthermore, there has been publication bias.

## 5. Conclusion

In conclusion, our review summarizes that a large proportion of the Ethiopian population have MetS regardless of the study subjects, gender, and the definitions used. One fact is clear here. MetS is growing at an alarming rate and is high in Ethiopia. Therefore, policymakers, clinicians, and concerned stakeholders shall urge effective strategies in the control, prevention, and management of MetS.

## Figures and Tables

**Table 1:** Definitions of metabolic syndrome by using NCEP/ATPIII and IDF criteria.

	NCEP/ATPIII	IDF
Absolutely required	None	Central obesity (waist circumference§): 94 cm (M) and ≥80 cm (F)
Criteria	Any three of the five criteria below	Obesity, plus two of the four criteria below
Obesity	Waist circumference: >40 inches (M) and >35 inches (F)	Central obesity already required
Hyperglycemia	Fasting glucose >100 mg/dl or Rx	Fasting glucose> 100 mg/dl
Dyslipidemia	TG > 150 mg/dl or Rx	TG > 150 mg/dl or Rx
Dyslipidemia (second, separate criteria)	HDL-C: <40 mg/dl (M) and <50 mg/dl (F) or Rx	HDL-C: <40 mg/dl (M) and <50 mg/dl (F) or Rx
Hypertension	>130 mmHg systolic or > 85 mmHg diastolic or Rx	>130 mmHg systolic or > 85 mmHg diastolic or Rx

Rx, pharmacologic treatment; NCEP/ATPIII, National Cholesterol Education Program–Adult Treatment Panel III; IDF, International Diabetes Federation; TG, triglyceride; HDL-C, high-density lipoprotein cholesterol.

**Table 2:** Methodological quality assessment of included studies using Joanna Brigg's Institute quality appraisal criteria scale (JBI).

Study [Ref.]/Year	Was the sample representative of the target population?	Were study participants recruited in an appropriate way?	Was the sample size adequate?	Were the study subjects and setting described in detail?	Was the data analysis conducted with sufficient coverage of the identified sample?	Were objective standard criteria used for measurement of the condition?	Was the condition measured reliably?	Are all the important confounding factors/subgroups/differences identified and accounted for?
Mossie et al. [36]/2016	Yes	Yes	Yes	Yes	Yes	Yes	Yes	Yes
Tachebele et al. [37]/2014	Yes	Yes	Yes	Yes	Yes	Yes	Yes	Yes
Bosho et al. [38]/2018	Yes	Yes	Yes	Yes	Yes	Yes	Yes	Yes
Hirigo et al. [39]/2016	Yes	Yes	Yes	Yes	Yes	Yes	Yes	Yes
Tran et al. [40]/2011	Yes	Yes	Yes	Yes	Yes	Not clear	Yes	Yes
Tesfaye et al. [41]/2014	Yes	Yes	Yes	Yes	Yes	Yes	Yes	Yes
Biadgo et al. [42]/2018	Yes	Yes	Yes	Yes	Yes	Yes	Yes	Yes
Birarra et al. [43]/2018	Yes	Yes	Yes	Yes	Yes	Yes	Yes	Yes
Solomon et al. [44]/2020	Yes	Not clear	Yes	Yes	Yes	Yes	Yes	Yes
Gebremeskel et al. [45]/2019	Yes	Yes	Yes	Yes	Yes	Yes	Yes	Yes
Bune et al. [46]/2020	Yes	Yes	Yes	Yes	Yes	Yes	Yes	Yes
Gebremariam et al. [47]/2020	Yes	Yes	Yes	Yes	Not clear	Yes	Yes	Yes
Asaye et al. [48]/2020	Yes	Yes	Yes	Yes	Yes	Yes	Yes	Yes
Wube et al. [49]/2019	Yes	Yes	Yes	Yes	Yes	Yes	Yes	Yes
Teshome et al. [50]/2020	Yes	Yes	Yes	Yes	Yes	Yes	Yes	Yes
Gebreyes et al. [51]/2018	Yes	Not clear	Yes	Yes	Yes	Not clear	Yes	Yes
Kerie et al. [52]/2019	Yes	Yes	Yes	Yes	Yes	Yes	Yes	Yes
Gebrehiwot et al. [53]/2020	Yes	Yes	Yes	Yes	Yes	Yes	Yes	Yes
Ataro et al. [54]/2020	Yes	Yes	Yes	Yes	Yes	Yes	Yes	Yes
Melak et al. [55]/2016	Yes	Yes	Yes	Yes	Yes	Yes	Yes	Not clear
Geto et al. [56]/2018	Yes	Yes	Yes	Yes	Yes	Yes	Yes	Yes

**Table 3:** Baseline characteristics and outcomes of the included studies (n = 28).

Study [Ref.]/Year	Region	Sampling techniques	Study area	Study subjects	Sample Size, *n*	*P*% (IDF)	*P*% (NCEP/ATPIII)	Quality score by JBI criteria
Berhan et al. [57]/2012	Oromia	MPSSS	Jimma	HIV on HAART	313	—	21.1	Good
Mossie et al. [36]/2016	Oromia	MPSSS	Jimma	Working adults	1316	16.7	10.5	Good
Tachebele et al. [37]/2014	Amhara	SRST	Gondar	Hypertensive	300	39.3	40.7	Good
Tadewos et al. [58]/2018	SNNP	SRST	Hawassa	Hypertensive	238	—	48.7	Good
Woyesa et al. [59]/2017	SNNP	SRST	Hawassa	T2DM patients	314	—	70.1	Good
Bosho et al. [38]/2018	Oromia	SRST	Jimma	HIV on HAART	268	20.5	23.5	Good
Hirigo et al. [39]/2016	SNNP	NA	Hawassa	HIV on HAART	185	24.3	17.8	Good
Tran et al. [40]/2011	Addis Ababa	MPSSS	Addis Ababa	Working adults	1935	17.9	12.5	Good
Abda et al. [60]/2016	Oromia	CST	Jimma	Working adults	225	—	26	Good
Tadewos et al. [61]/2017	SNNP	SRST	Hawassa	T2DM patients	270	—	45.9	Good
Tesfaye et al. [41]/2014	SNNP	SRST	Hawassa	HIV on HAART	188	25	18.1	Good
Biadgo et al. [42]/2018	Amhara	SRST	Gondar	T2DM patients	159	53.5	66.7	Good
Birarra et al. [43]/2018	Amhara	SRST	Gondar	T2DM patients	256	57	70.3	Good
Solomon et al. [44]/2020	Addis Ababa	NA	Addis Ababa	Working adults	325	8.6	20.3	Good
Gebremeskel et al. [45]/2019	Tigray	SRST	Mekele	T2DM patients	419	51.1	—	Good
Bune et al. [46]/2020	SNNP	SRST	Gedeo zone	HIV on HAART	422	22	—	Good
Gebremariam et al. [47]/2020	Tigray	Survey	Mekele	Working adults	1380	40.7	—	Good
Asaye et al. [48]/2020	Oromia	CST	Jimma	Psychiatric patients	360	28.9	22.2	Good
Wube et al. [49]/2019	SNNP	SRST	Hawassa	T2DM patients	314	52.9	70.1	Good
Teshome et al. [50]/2020	SNNP	SRST	Hawassa	Psychiatric patients	245	26.9	24.5	Good
Zerga et al. [62]/2020	Amhara	CST	Desie	T2DM patients	330	-	59.4	Good
Gebreyes et al. [51]/2018	All regions	NA	—	Working adults	8673	4.8	—	Good
KerieS et al. [52]/2019	SNNP	SRST	Mizan-Aman	Working adults	534	9.6	—	Good
Gebrehiwot et al. [53]/2020	Tigray	SRST	Mekele	Working adults	266	21.8	—	Good
Ataro et al. [54]/2020	Eastern Ethiopia	CST	Harar	HIV on HAART	375	26.7	22.1	Good
Cheneke et al. [63]/2019	Eastern Ethiopia	CST	Harar	Working adults	365	-	27.7	Good
Melak et al. [55]/2016	Amhara	SRST	Gondar	Working adults	227	13.6	—	Good
Geto et al. [56]/2018	Addis Ababa	CST	Addis Ababa	Working adults	450	27.6	16.7	Good

NCEP/ATPIII, National Cholesterol Education Program–Adult Treatment Panel III; IDF, International Diabetes Federation; T2DM, type 2 diabetes mellitus; MPSSS, multistage probabilistic stratified sampling strategy; SRST, simple/systematic random sampling technique; CST, consecutive sampling technique; SNNP, South Nation Nationality and People; HAART, highly active antiretroviral therapy; JBI, Joanna Brigg's Institute quality appraisal.

**Table 4:** Pooled estimates of metabolic syndrome based on sex/gender.

Study/Year	Sample size (male)	Sample size (female)	*P*% male by NCEP/ATP III	*P*% female by NCEP/ATP III	*P*% male by IDF	*P*% female by IDF
Berhan et al. [57]/2012	109	204	17.4	23	—	—
Mossie et al. [36]/2016	620	696	6	14.5	11.1	21.7
Tachebele et al. [37]/2014	115	185	12	28.7	9.3	30.3
Tadewos et al. [58]/2018	105	133	37.9	62.1	—	—
Bosho et al. [38]/2018	57	211	21	24.2	—	—
Hirigo et al. [39]/2016	68	117	17.6	17.9	8.8	33.3
Tran et al. [40]/2011	1171	764	10	16.2	—	—
Abda et al. [60]/2016	106	119	16	35.3	14	24
Tadewos et al. [61]/2017	166	104	36.7	60.6	—	—
Tesfaye et al. [41]/2014	126	248	18.3	16.1	15.1	28.2
Biadgo et al. [42]/2018	64	95	56.3	73.7	—	—
Birarra et al. [43]/2018	113	143	33.9	66.1	27.4	72.6
Solomon et al. [44]/2020	155	170	18.1	22.4	1.9	14.7
Gebremeskel et al. [45]/2019	208	211	—	—	42.5	57.5
Gebremariam et al. [47]/2020	823	557	—	—	41.1	40.2
Asaye et al. [48]/2020	229	131	11.4	17.5	—	—
Wube et al. [49]/2019	211	103	69.7	70.9	32.7	94.2
Teshome et al. [50]/2020	143	102	46.7	53.3	47	53
Zerga et al. [62]/2020	170	160	—	—	—	—
Gebreyes et al. [51]/2018	1306	7367	—	—	8.6	1.8
KerieS et al. [52]/2019	271	263	6.6	12.5	—	—
Gebrehiwot et al. [53]/2020	124	142	—	—	18.5	24.6
Cheneke et al. [63]/2019	—	365	−202	27.7	—	—
Geto et al. [56]/2018	232	218	19.4	13.8	35.8	18.8
Combined pooled estimate (95% CI: LCI, UCI)	24.82% (95% CI: 18.34, 31.31)	34.09% (95% CI: 26.68, 41.50)	22.22% (95% CI: 14.89, 29.56)	36.74% (95% CI: 20.72, 52.75)		

NCEP/ATPIII, National Cholesterol Education Program–Adult Treatment Panel III; IDF, International Diabetes Federation; LCI, lower confidence interval; UCI, upper confidence interval.

**Table 5:** Pooled estimates of metabolic syndrome components.

Authors	Sample size	Prevalence of metabolic syndrome components				
		Hypertension	Hyperglycemia	Low HDL-C	Central obesity	Elevated triglyceride
Berhan et al. [57]	313	16	24.9	46	13.7	39
Tachebele et al. [37]	300	—	25.3	81.3	18.7	27.3
Tadewos et al. [58]	238	—	35.7	60.9	37.8	62.2
Woyesa et al. [59]	319	25.5	80	39.2	61.3	70.4
Bosho et al. [38]	268	38.4	17.2	49.3	18.7	29.9
Hirigo et al. [39]	185	9.72	14.59	70.27	22.7	44.86
Tran et al. [40]	1935	20.65	19.97	23.32	18.5	20.59
Abda et al. [60]	225	45	39	31	26	18
Tadewos et al. [61]	270	28.1	—	47	40.7	68.1
Esfaye et al. [41]	188	23.9	33.5	53.7	52.7	45.2
Biadgo et al. [42]	159	55.4	—	32.7	43.4	56.6
Melak et al. [55]	220	15.5	8.6	68.6	17.7	26.8
Ataro et al. [54]	375	10.9	18.4	64.5	59.2	41.9
Birarra et al. [43]	256	43	—	66.8	16.4	67.6
Bune et al. [46]	422	56.6	56.4	30.8	49.1	42.9
Gebremariam et al. [47]	1380	19.6	19.4	71.6	42.7	55.7
Gebremeskel et al. [45]	419	41.3	—	34.4	59.7	45.1
Gebreyes et al. [51]	8673	15.8	9.1	68.7	39.8	21.0
Geto et al. [56]	450	23.6	2.4	41.3	80.2	19.3
Kerie S et al. [52]	534	16.9	7.1	50.9	26.22	22.8
Solomon S et al. [44]	325	36.3	32.6	48.6	19.4	24.0
Teshome T et al. [50]	245	22.0	34.3	41.2	24.9	26.9
Combined pooled estimates (LCI, UCI)		**27.87 (23.4, 32.2)**	**26.4 (20, 32.8)**	**51.0 (42.4, 59.7)**	**35.85 (28.9, 42.8)**	**39.7 (32.8, 46.6)**

HDL-C, high-density lipoprotein cholesterol; LCI, lower confidence interval; UCI, upper confidence interval.

## Data Availability

The data used to support the findings of this study are available from the corresponding author upon request.

## Abbreviations

CVD: Cardiovascular diseases
IDF: International Diabetes Federation
HAART: Highly active antiretroviral therapy
MetS: Metabolic syndrome
NCEP/ATP III: National Cholesterol Education Program–Adult Treatment Panel III (NCEP/ATP III).
